# The FtsZ Homolog, FszB, Inhibits Mitochondrial Dynamics in *Dictyostelium discoideum*

**DOI:** 10.3390/cells9010064

**Published:** 2019-12-25

**Authors:** Ericka Vogel, Pristine Bay Pittman, Kari Naylor

**Affiliations:** Department of Biology, University of Central Arkansas, Conway, AR 72035, USA; Ericka.vogel@aurora.org (E.V.); pristinepittman@yahoo.com (P.B.P.)

**Keywords:** mitochondrial dynamics, fission, fusion, motility, cytoskeleton, actin, microtubules

## Abstract

*Dictyostelium discoideum* is a well-established mitochondrial model system for both disease and dynamics, yet we still do not understand the actual mechanism of mitochondrial dynamics in this system. The FtsZ proteins are known to mediate membrane remodeling events such as cytokinesis in bacteria and fission of chloroplasts; *D. discoideum* has two FtsZ proteins, FszA and FszB. To determine the role of these proteins in mitochondrial dynamics we overexpressed FszB-GFP and determined its effect on fission, fusion, and motility in the presence of intact and disrupted cytoskeletal filaments. Here we show that overexpression of FszB-GFP decreases mitochondrial dynamics and suggest that actin may play a positive role driving fission in the context of excessive inhibition by overexpressed FszB-GFP.

## 1. Introduction

Mitochondria play a central role in the function of normal, healthy cells. Beyond its dominant role as a producer of ATP via oxidative phosphorylation, mitochondria also mediate metabolic functions such as the generation of reactive oxidative species (ROS), regulation of cell signaling, cell death, and biosynthetic metabolism [1]. These critical functions are reliant on the appropriate mitochondrial structure, which in turn, is dependent on mitochondrial dynamics including fission, fusion, and motility. Fusion is the process by which mitochondria join together, whereas fission is the process that allows the separation of mitochondria [2]. Fission facilitates the distribution of mitochondria along cytoskeleton tracts, which provides energy throughout the cell [3], and helps to sequester damaged mitochondria and promote autophagy of these damaged organelles [4,5]. Mitochondrial fission is also required for apoptosis, where cells with excessive damage undergo programmed cell death [6,7]. In comparison, fusion supports mitochondrial function as it provides a way for mitochondria to mix their contents, allowing protein complementation, mitochondrial DNA repair, and distribution of metabolites [8,9]. Fusion is also a necessary component of apoptosis [6,7]. As a result of fission and fusion, these organelles are continuously altering in shape, size, and number throughout the life of a cell [10]. In addition to the dynamic processes themselves, mitochondrial morphology itself may predict fission or fusion events. Features such as perimeter and clustering are positively correlated with fission and fusion, respectively. These parameters are consistent with the observation that large mitochondria with an expansive perimeter have a greater tendency to divide, whereas fusion events are more likely to occur when there is the least distance to a neighboring mitochondrion [11].

Mitochondria must be mobile in order to maintain fission and fusion events. Alternatively, loss of fission and fusion has been shown to impede motility and distribution of mitochondria within the cell [8,12], while other studies have shown that non motile mitochondria become shorter [13], suggesting that fission and fusion promote motility. Furthermore, our previous work has demonstrated that microtubules are key to the movement and distribution of mitochondria in our model organism, *Dictyostelium discoideum* as well as fission and fusion [14]. In neurons, similar results have been found, where mitochondrial dynamics are disrupted when the cytoskeleton is altered [15,16]. It is clear that these dynamics are very interdependent upon each other.

These interdependent mitochondrial dynamics and mitochondrial morphology are an indicator of a healthy, properly functioning organelle and cell [8,17,18,19]. In particular, when these functions are disrupted, neurons can be affected. It is known that neurons have high-energy demands and can be affected by disruptions in the mitochondria [17]. In particular, disruption of mitochondrial dynamics can cause or contribute to neurodegenerative diseases such as Dominant Optic Atrophy (childhood blindness), Parkinson’s disease, and Alzheimer disease [8,19,20,21,22,23].

To better understand how mitochondrial dynamics contribute to neurodegeneration, we use the simple model system, *D. discoideum*, a social amoeba, that offers advantages as a model organism as many of its genes are homologous to human genes [24]. This organism is evolutionarily unique in that it sits between uni- and multicellularity and can thereby be used to elucidate many processes that are too difficult to observe in higher organisms [25]. Thus, *D. discoideum* is the perfect system to understand mitochondrial dynamics and contribute to understanding the role of mitochondrial dynamics in a variety of diseases. [26,27,28].

As theory suggests, mitochondria evolutionarily originate from free-living bacteria, or ɑ-proteobacteria. In bacteria, cell division is driven by the tubulin homologue, FtsZ. FtsZ forms a Z-ring that provides scaffolding for the assembly of bacterial cytokinetic machinery on the inside of the cell [29,30]. This ring will constrict, driving the cell through cytokinesis. Mitochondria in higher eukaryotes (such as mammals) have lost the FtsZ proteins using an alternative protein family: Dynamin-related proteins (DRP’s) for division. Lower eukaryotes and chloroplasts have retained the FtsZ family [31,32,33,34,35]. Since both chloroplast and mitochondria are believed to have evolved through a similar process, it is logical that mitochondria also have their own unique FtsZ homologs that function in membrane dynamics.

*D. discoideum* expresses two members of the FtsZ family, FszA and FszB, which are thought to be involved in mitochondrial dynamics [32]. In order to better understand the role of FtsZs in mitochondrial dynamics we began with overexpressed FszB, fused to a GFP tag, and quantified mitochondrial dynamics by determining rates of fission, fusion, and motility. We analyzed the localization of FszB-GFP during fission and fusion events to further understand the role of this possible protein filament on mitochondrial dynamics. Due to the involvement of the cytoskeleton in fission, fusion, and motility [14], we also analyzed these dynamics under conditions overexpressing FszB-GFP and a disrupted cytoskeleton. Understanding the molecular mechanism of mitochondrial dynamics can establish mitochondrial dynamics as a paradigm in humans, providing insight into neurodegenerative diseases and mitochondrial-related disorders.

## 2. Materials and Methods

### 2.1. Creation of FszB-GFP Strain

To create the FszB-GFP expressing strain, the FszB-GFP plasmid, pDXA3c with Act15 promotor, [32] was transformed into AX4 cells [36], using a Bio-Rad Xcell electroporator (Hercules, CA). The Bio-Rad Xcell electroporator was set to its exponential setting, 420 volts, 325 μF, and 2 pulses, 5 s apart. AX4 cells at a concentration of 3 × 10^6^ cells/mL were washed four times with electroporation buffer (50 mM sucrose, 16.5 mM KH_2_PO_4_, 3.9 mM K_2_HPO_4_, 2 mM MgSO_4_ × 7H_2_O) to ensure no extra salts were present. Varying amounts of DNA were added no more than 10% of the total volume of cells can be DNA to prevent electrical arcing. Once electroporated, cells were transferred to a petri dish and observed for 1–2 weeks for growth at 22 °C. Twenty-four hours after electroporation we added 10 µg/mL G418 (Thermo Fisher Scientific Grand Island, NY, USA) to select for cells containing the plasmid. Media was changed every 4 days while waiting for cells to grow. Once visible growth was present, the cells were resuspended from the plates in fresh media in an Erlenmeyer flask at a concentration of 4 × 10^4^ cells/mL, continuing selection. Once cells reach 1 × 10^6^ cells/mL, the cells were diluted and were made into permanent stocks.

### 2.2. Imaging Mitochondrial Dynamics

Log phase cells were prepared for imaging by washing 1 time and resuspending in Lo-Flo (Formedium, Hunstanton, Norfolk, UK). Cells were stained with 0.1 µM final concentration of Mitotracker CMXRos, (Invitrogen, Thermo Fisher Scientific, Grand Island, NY, USA) washed 3 times with Lo-Flo and placed in Nunc Lab-TekII 4-well chambered coverglass for imaging. To image the treated cells, we utilized a Zeiss laser scanning LSM 880 confocal microscope (Zeiss, Stockholm, Sweden) with a pinhole setting of 144 µm. This allows for a 1.1 µm optical slice. Cells were imaged on a minimum of 3 separate occasions with approximately 5 time-lapse images obtained each time.

### 2.3. Disruption of Actin with Latrunculin-B

During the last 30 min of incubation with Mitotracker, 10 µM Latrunculin-B (Lat-B) (Sigma-Aldrich, St. Louis, MO, USA) or equivalent volume of EtOH were added to the cells. Cells were washed and imaged as described above.

### 2.4. Disruption of the Microtubules with Nocodazole

During the last hour of incubation with Mitotracker, 10 µg/mL nocodazole (Noc) (Sigma-Aldrich, St. Louis, MO, USA) or equivalent volume of DMSO were added to the cells. Cells were washed and imaged as described above.

### 2.5. Analyzing Morphology and Size

We classified the morphology of the mitochondria according to the size and distribution of the organelle and FszB-GFP complexes. Distribution classification was determined as appearing overexpressed, random, even, or clustered (similar to [26]). The overexpressed classification was used if components were not distinguishable as individuals but just excessive fluorescence. Random classification signified that there was no distinct pattern to the distribution of mitochondria in the cells. Mitochondria were classified as even if the distribution was throughout the cell, while clustered mitochondria and complexes were aggregated either near the periphery or center of the cell. Size was classified by looking at each cell and making a judgement on whether the majority of the mitochondria were small, medium, or large. Values were compared with Chi-Square (*n* = 50 across 3 replicates) using GraphPad 6.07 (La Jolla, CA, USA, www.graphpad.com), and *p*-values of less than 0.05 were considered statistically significant.

### 2.6. Kymographs and Quantifying Motility Rates

Motility was quantified using Fiji Open Source software (https://imagej.net/Fiji) to create kymographs. Regions of interest (ROIs) were drawn through the left, middle, and right sector of each cell. ROIs were then stacked and converted to create a kymograph to track mitochondrial movement. Kymographs were made for 50 cells; velocity was calculated and averaged for each ROI and then per cell as pixels/0.677 s and later converted to micrometers/second. Velocities of AX4 mitochondria were compared using Welch’s t-test GraphPad Prism 6.07 (La Jolla, CA, USA, www.graphpad.com). The averaged velocities for treated (Lat-B or Noc) samples were compared in JMP 14.0.0 (SAS Institute, Inc, Cary, NC, USA) using Kruskal-Wallis with Dunn’s post-hoc analysis (*n* = 50, across 3 replicates), *p*-values of less than 0.05 were considered statistically significant.

Average percent of mitochondria moving in 50 cells from 3 biological replicates was also calculated from the kymographs. Mitochondria were counted and classified as moving or not moving. Non-moving components produce a straight, vertical line that spanned the length of the kymograph. The number of moving/non-moving mitochondria were averaged for each strain and compared using GraphPad Prism 6.07 (La Jolla, CA, USA, www.graphpad.com) Mann–Whitney analysis for untreated cells or JMP 14.0.0 (SAS Institute, Inc, Cary, NC, USA) Kruskal–Wallis with Dunn’s post hoc, *p*-values of less than 0.05 were considered statistically significant.

### 2.7. Counting Rates of Fission, Fusion

Image analysis of fission and fusion was performed with Zen 2.1 software (Zeiss, Stockholm, Sweden, https://www.zeiss.com/microscopy/us/products/microscope-software/zen-lite.html). Fission was determined by a single organelle splitting into two organelles. Fusion was quantified by two mitochondria nearing each other for multiple frames and then fusing into one organelle. The rates of fission and fusion were determined by calculating the average number of events/min./cell for a minimum of 30 cells in each strain and treatment, across 3 replicates. Rates were compared using Kruskal–Wallis with Dunn’s post-hoc analysis via JMP 14.0.0 (SAS Institute, Inc, Cary, NC, USA). A *p*-value of less than 0.05 was considered statistically significant.

### 2.8. Determining FszB Localization

FszB localization was determined through the use of Zen 2.1 software (Zeiss, Stockholm, Sweden, https://www.zeiss.com/microscopy/us/products/microscope-software/zen-lite.html). A fission or fusion event would first be identified in the Mitotracker Red field of vision. Then, the fluorescent green field of vision would be utilized. Once the FszB-GFP expression is visible, the localization of FszB was determined during the fission or fusion event. FszB presence or absence was then recorded. This was done for every identified fission/fusion event, *n* = 30 cells, across 3 replicates. Statistical analysis was done via Wilcoxon Match Pairs Signed Rank test by GraphPad Prism 6.07 (La Jolla, CA, USA, www.graphpad.com), a *p*-value less than 0.05 was considered statistically significant.

## 3. Results

### 3.1. FszB-GFP Overexpression Does Not Change Mitochondrial Distribution nor Size

**Figure 1:**
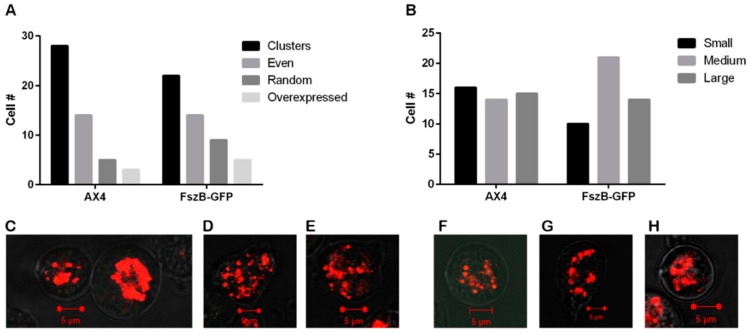
Distribution and size of mitochondria in AX4 and FszB-GFP cells. (**A**) There is no significant difference between the 38% of AX4 cells and 46% of FszB-GFP cells that have evenly or randomly distributed mitochondria (n = 50). (**B**) There is also no statistical difference between cell types when roughly estimating the size of the mitochondria (n = 50 cells). (**C**) Example image of clustered and overexpressed mitochondria. Left cell shows clustered phenotype, right cell shows an overexpressed phenotype. (**D**) Example image of evenly distributed mitochondria and (**E**) example of randomly distributed mitochondria- neither truly aggregated nor fully distributed across the cell.

Many organisms have tubular highly interconnected mitochondria [37] yet *D. discoideum* mitochondria demonstrate a fragmented phenotype even though fission and fusion are balanced events [28]. To determine if overexpression of FszB-GFP alters this fragmented phenotype we quantified mitochondria shape and distribution in wild-type (AX4) and FszB-GFP overexpressing cells. Our results indicate that 38% of wild-type cells have evenly or randomly distributed mitochondria which is not significantly different from the 46% of FszB-GFP cells with this phenotype (Figure 1A). Additionally, there is no difference in mitochondrial size (Figure 1B).

### 3.2. Overexpressed FszB-GFP Inhibits Mitochondrial Dynamics but Localizes to Fission and Fusion Events

**Figure 2:**
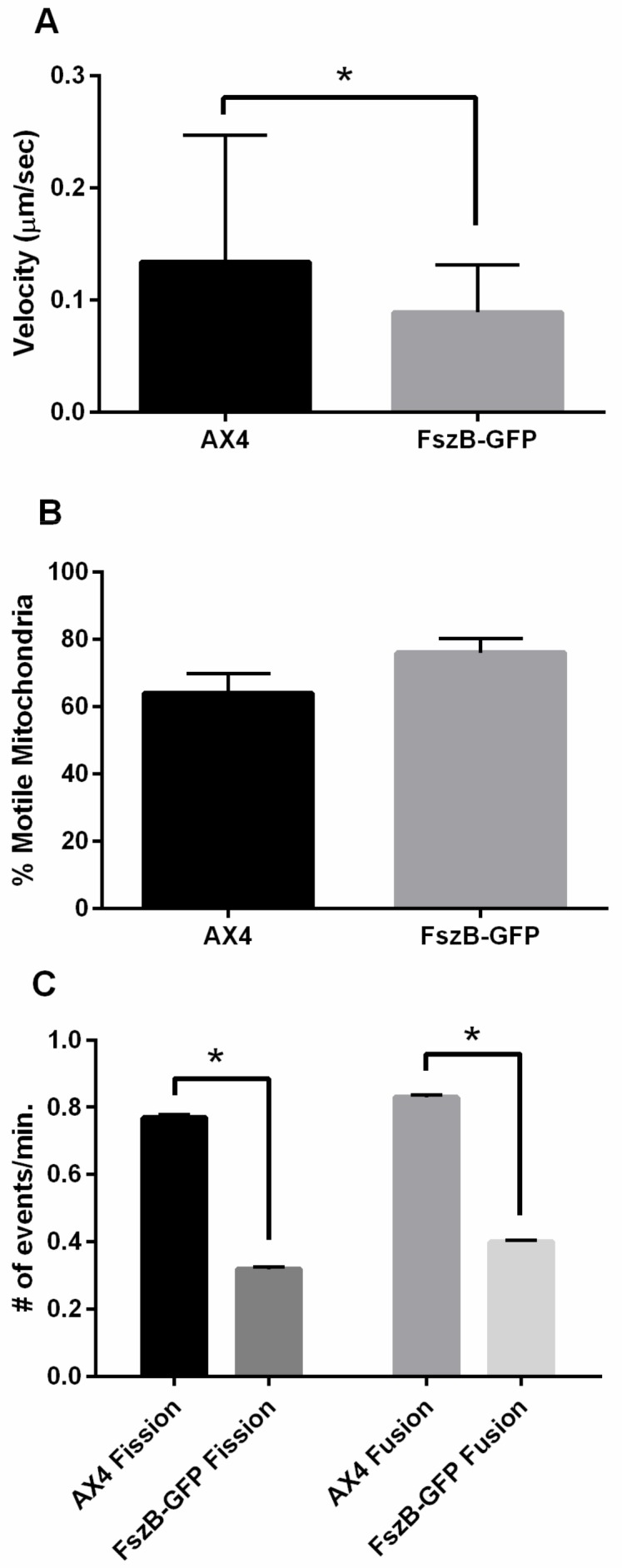
Average velocity, percent motility, fission, and fusion rates in AX4 and overexpressed FszB-GFP cells. (**A**) In overexpressed FszB-GFP cells, velocity was slower than AX4 by 51% (*p* = 0.0081). (**B**) Overexpression of FszB-GFP has no effect on the percentage of mitochondria that are motile, (**C**) while overexpression of FszB-GFP does significantly decrease fission (*p* < 0.0001) and fusion (*p* < 0.0001). *n* = 50 cells for (**A**) and (**B**), *n* = 30 cells for (**C**), * indicates significance.

To determine the role of FszB-GFP in mitochondrial dynamics we quantified mitochondrial fission, fusion, motility, and localization in wild-type and cells overexpressing the FszB-GFP construct. Previously published work found that wild-type cells (AX4) had an average mitochondrial velocity of 0.164 ± 0.007 µm/sec [14]. In the current study we similarly found that AX4 mitochondria move at a rate of 0.134 ± 0.016 µm/sec, but mitochondria in cells overexpressing FszB-GFP move approximately 51% more slowly at a rate of 0.089 ± 0.06 µm/sec in comparison to AX4 mitochondria (*p* = 0.0081) (Figure 2A).

From the kymographs created for motility analysis, the percent of mitochondrial movement was also calculated. An average of 64 ± 5.8% of AX4 mitochondria are moving with an average of 76 ± 4.2% of FszB-GFP mitochondria are moving (Figure 2B), there is no statistical difference between AX4 and FszB-GFP mitochondria in terms of number of moving organelles.

We determined the rates of fission and fusion in wild-type and FszB-GFP cells to determine if overexpression of FszB alters these processes (Video S1,S2). Wild-type cells undergo fission at a rate of 0.77 ± 0.009 events/min and fusion at a rate of 0.83 ± 0.007 events/min. (Figure 2C). Mitochondria overexpressing FszB-GFP undergo fission at 0.32 ± 0.006 events/min. and fusion at 0.4 ± 0.006 events/min (Figure 2C). These rates are significantly decreased for both dynamics (*p* < 0.0001 for both).

To determine why FszB-GFP decreases fission and fusion we analyzed localization. It has been established that FszB-GFP localizes to the mitochondria [32]. Here we analyzed fission and fusion events to determine whether or not FszB-GFP was present during these events (Supplementary Video 2). We demonstrate that FszB-GFP significantly localizes to 100% of the observed fusion events and 90% of the observed fission events (*p* < 0.0001 for both).

When considering fission, fusion, and motility, FszB-GFP overexpression significantly decreases these rates from wild-type cells, though it has no effect on percent motile mitochondria. Since FszB-GFP is significantly localized to the sites of these events it is quite possible that FszB-GFP is a negative regulator of these processes.

### 3.3. Mitochondrial Motility Is Decreased with FszB-GFP and Disrupted Actin Cytoskeleton

We have previously shown that the cytoskeleton has significant effects on mitochondrial dynamics, specifically disruption of the actin cytoskeleton decreased the percent of motile mitochondrial. Contrarily, disruption of the microtubule cytoskeleton decreased fission, fusion, and velocity of mitochondria movement [14]. To determine if FszB-GFP has an interaction with the cytoskeleton, we treated cells with Latrunculin-B or nocodazole and quantified mitochondrial dynamics when FszB-GFP was overexpressed.

**Figure 3:**
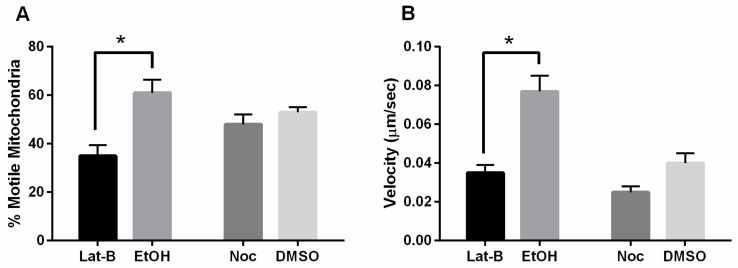
Percent motility and velocity of mitochondria in cells overexpressing FszB-GFP and disrupted cytoskeleton. (**A**) Disruption of the actin cytoskeleton decreases the percent of motile mitochondria (*p* = 0.0016), while disruption of the microtubules has little effect. (**B**) Disruption of the actin cytoskeleton significantly decreases velocity (*p* = 0.0005). *n* = 50 cells, * indicates significance.

Similar to the Woods et al., 2016 study, we found that the percent motile mitochondria in cells overexpressing FszB-GFP when treated with Lat-B decreased by 74% (*p* = 0.0016) [16]. There was no significant difference in percent motility of mitochondria when treated with Noc (Figure 3A). Analysis of mitochondrial velocity shows that cells overexpressing FszB-GFP and treated with either Lat-B or Noc, have a decreased mitochondrial velocity. Lat-B mitochondrial velocity decreased by 120% compared to the vehicle control (*p* = 0.0005), while the Noc treatment decreased velocity by 64%; though, this was not significant (Figure 3B).

In summary, disruption of the actin cytoskeleton and overexpression of FszB-GFP decreases the percent motile mitochondria and the speed at which these organelles move, whereas Lat-B alone only decreases percent motile mitochondria [14] and FszB-GFP overexpression alone only decreased mitochondrial velocity. On the other hand, treatment with Noc in FszB-GFP overexpressing cells does not alter percent motility and only slightly decreases velocity of mitochondria compared to vehicle whereas Noc alone significantly decreases velocity.

### 3.4. Disruption of the Actin Cytoskeleton When FszB-GFP Is Overexpressed Unbalances Fission and Fusion

**Figure 4:**
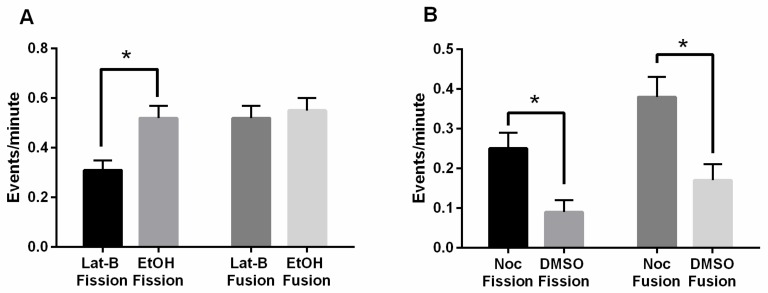
Fission and fusion in treated cells overexpressing FszB-GFP. (**A**) Treatment with Lat-B in comparison to EtOH decreases fission events by 68% (*p* = 0.0240), there is no difference in fusion. (**B**) Noc treatment increases fission, 64%, and fusion, 55%, in comparison to the vehicle (*p* = 0.0150, 0.0002 respectively). These rates are similar to untreated cells overexpressing FszB-GFP. *n* = 30 cells, * indicates significance.

To again explore the possible connection between the cytoskeleton and FszB, we quantified fission and fusion under Lat-B and Noc treatments and compared the rates to the respective vehicles. Our previous data showed that in AX4 cells treated with Lat-B, fission and fusion rates were not significantly affected, though inhibition of microtubules significantly decreased these rates (16). In this study, mitochondria overexpressing FszB-GFP and treated with Lat-B undergo fission at a rate of 0.31 ± 0.04 events/min, while vehicle cells undergo fission at a rate of 0.52 ± 0.05 events/min. (Figure 4A). This is a significant decrease of 68% in fission rates for mitochondria treated with Lat-B, (*p* = 0.0240). The fusion rate for mitochondria treated with Lat-B is 0.52 ± 0.05 events/min., and the vehicle cells undergo a similar fusion rate of 0.55 ± 0.05 events/min. (Figure 4A).

In the presence of Noc, or microtubule inhibition, the fission rate of 0.25 ± 0.04 events/min. was 64% faster than the vehicle rate of fission was 0.09 ± 0.03 events/min, (*p* = 0.0150) (Figure 4B). Mitochondria treated with Noc underwent fusion at a rate of 0.38 ± 0.05 events/min. compared to the vehicle fusion rate of 0.17 ± 0.04 events/min. This is a significant increase, by 55%, in fusion in cells treated with Noc and overexpressing FszB-GFP (*p* = 0.0002). These trends indicate fission and fusion become unbalanced when FszB-GFP is overexpressed and the actin cytoskeleton is disrupted; specifically, fission decreases while fusion remains the same. Interestingly, when microtubules are disrupted with Noc both fission and fusion are increased compared to the vehicle DMSO.

To determine if any of these changes are caused by a change in the localization pattern of FszB-GFP we again determined the presence or absence of FszB-GFP during fission and fusion events (Table 1). Our data indicated that only DMSO alters the localization of FszB-GFP during fusion events. Only 82% of fusion events have FszB-GFP present suggesting that some fusion events may take place without a detectable FszB (*p* = 0.0557).

### 3.5. Disruption of the Cytoskeleton and Overexpressing FszB-GFP Does Not Alter Morphology or Size

**Figure 5:**
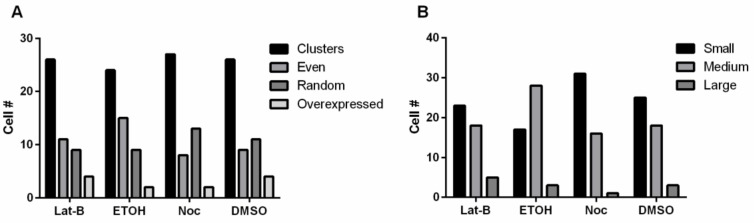
Analysis of mitochondrial distribution and size when treated with Lat-B or Noc. Disruption of the cytoskeleton does not alter (**A**) distribution or (**B**) size of the mitochondria when FszB-GFP is overexpressed. *n* = 50 cells.

We have previously shown that disrupting the cytoskeleton does not affect mitochondrial morphology [16]. Here we wanted to determine if overexpressing FszB-GFP and disrupting the cytoskeleton might change this result. Thus, we quantified mitochondrial distribution and size after treating the cells with Lat-B or Noc. Overall, we found that these treatments had little effect on mitochondrial size and distribution with the disruption of the cytoskeleton (Figure 5).

## 4. Discussion

### 4.1. FszB-GFP Inhibits Fission, Fusion, and Motility

In many organisms, the morphology of mitochondria indicates functionality of fission and fusion processes. For example, in yeast, worms, or mammals, loss of Drp-1 prevents fission causing the mitochondria to become a highly interconnected network [35], while overexpression of Drp-1 increases fission causing fragmentation [38,39]. To quickly determine if FszB-GFP overexpression in AX4 cells has any effect on dynamics we analyzed mitochondrial morphology and distribution and found no alteration suggesting that FszB-GFP overexpression does not affect mitochondrial dynamics in this cell background. Interestingly, by carefully counting fission and fusion events and measuring mitochondrial velocity, we show that excess FszB-GFP does significantly decrease motility, fission and fusion.

One reason for this decrease in mitochondrial dynamics may be that the overexpression of FszB-GFP may block the interaction of the mitochondria with the cytoskeleton. Woods et al., shows that loss of microtubules decreases the speed of mitochondrial movement, while loss of actin filaments decreases the percent of mitochondria that are motile in *D. discoideum* [14]. Overexpression of FszB-GFP does not alter the percent of motile mitochondria suggesting that the defect lies with the interaction with the microtubules. An alternative hypothesis is that mitochondrial fission, fusion, and motility are interdependent [8,12,14], thus it is possible that overexpression of FszB-GFP alters the interdependence of these processes. FszB-GFP is present at 90–100% of fission and fusion events, perhaps the excess of FszB prevents fission and fusion, which subsequently decreases motility of the organelles that are actively attempting to undergo fission and/or fusion.

### 4.2. Actin Counteracts FszB-GFP to Mediate Fission Events

To begin to distinguish between the possibilities, discussed above, as to why overexpressed FszB-GFP inhibits fission, fusion, and speed of mitochondrial movement we studied mitochondrial dynamics when the cytoskeleton was disrupted and FszB-GFP overexpressed. Using Lat-B to disrupt the actin filaments we see that the speed and percent of motile mitochondria is decreased. Lat-B alone decreases organelles that are motile [14] while overexpressed FszB-GFP alone decreases speed. The combination of treatments results in a combination of the phenotypes suggesting independent effects from the treatments and that actin filaments are not involved in speed of mitochondria and FszB is not involved in the loading of mitochondria onto the microtubules.

Analysis of fission and fusion in FszB-GFP overexpressing cells treated with Lat-B demonstrates an unbalancing of fission and fusion events. Overexpression of FszB-GFP decreases fission and fusion, while treatment with Lat-B alone has no effect [14], the combination of treatments results in a decrease of fission by 68%, even though FszB-GFP still localizes to fission and fusion events 83–89% of the time. Perhaps actin is working to overcome the inhibition of fission by FszB-GFP and when actin is disrupted the mitochondria receive the full negative effect of overexpressed FszB-GFP. In mammalian cells the endoplasmic reticulum (ER) marks future fission sites and actin polymerization promotes fission and inhibits fusion [40,41,42]. Thus, future work needs to more closely study the effect of actin and potentially the ER on mitochondrial dynamics in *D. discoideum* cells.

### 4.3. Microtubules and FszB-GFP Mediate Opposing Function in the Process of Mitochondrial Motility

When cells overexpressing FszB-GFP are treated with Noc there is no effect on motility, despite the individual treatments both decreasing mitochondrial velocity. This suggests that loss of microtubules and overexpressed FszB-GFP neutralize each other’s role in terms of mitochondrial velocity. Fission and fusion rates under these same conditions are increased with FszB-GFP still localizing to the mitochondria 89–97% of the time. Without motility, the mitochondria do not have the necessary movement that would allow them to interact and fuse together or upon division to fully separate [12]. In fact, once their dynamics have been disrupted, the movement of mitochondria in mammalian cells is more reminiscent of Brownian motion—i.e., they lack directed movement [12]. Perhaps once motility is restored in cells overexpressing FszB-GFP and treated with Noc, the intact actin cytoskeleton plays a larger role in driving fission and fusion events than previously thought.

## 5. Conclusions

As proposed in Woods et al., 2016, this work supports the hypothesis that *D. discoideum* actin filaments help mitochondria load onto microtubule tracks where the organelles can reach full velocity. We also propose that FszB-GFP is a direct negative inhibitor of fission and fusion; therefore when overexpressed, the mitochondria move slower because of the interdependence of these dynamics. Finally, we suggest that actin may have a positive role in *D. discoideum* mitochondrial fission. This work leads to a better understanding of the *D. discoideum* mechanism of mitochondrial dynamics and ultimately provides awareness to the evolution of the current mammalian mechanism of mitochondrial dynamics. Our results have the potential to improve knowledge of the role of mitochondrial dynamics play on mitochondrial dysfunction leading to disease.

## Figures and Tables

**Table 1:** Localization of FszB-GFP during fission and fusion events with an altered cytoskeleton. The table indicates % of events that FszB-GFP is present at. Statistically FszB-GFP is mis-localized during fusion events treated with DMSO, *p* = 0.0557, *n* = 30 cells.

Event	Lat-B	EtOH	Noc	DMSO
Fission	89%	100%	97%	100%
Fusion	83%	100%	89%	82%

## Supplementary Materials

The following are available online at https://www.mdpi.com/2073-4409/9/1/64/s1, Video S1: AX4 cells labeled with Mitotracker CMXRos; Video S2: AX4 cells expressing FszB-GFP and labeled with Mitotracker CMXRos.
